# Towards a Comprehensive Understanding of UA-ADRCs (Uncultured, Autologous, Fresh, Unmodified, Adipose Derived Regenerative Cells, Isolated at Point of Care) in Regenerative Medicine

**DOI:** 10.3390/cells9051097

**Published:** 2020-04-29

**Authors:** Eckhard U. Alt, Glenn Winnier, Alexander Haenel, Ralf Rothoerl, Oender Solakoglu, Christopher Alt, Christoph Schmitz

**Affiliations:** 1 Heart and Vascular Institute, Department of Medicine, Tulane University Health Science Center, New Orleans, LA 70112, USA; 2 Sanford Health, University of South Dakota, Sioux Falls, SD 57104, USA; 3 University of Texas MD Anderson Cancer Center, Houston, TX 77054, USA; 4 Isar Klinikum Munich, 80331 Munich, Germany; 5 InGeneron, Inc., Houston, TX 77054, USA; 6 Department of Radiology and Nuclear Medicine, University Hospital Schleswig-Holstein, 23562 Lübeck, Germany; 7 Dental Department of the University Medical Center Hamburg-Eppendorf, 20246 Hamburg, Germany; 8 Periodontology and Implant Dentistry, 22453 Hamburg, Germany; 9 InGeneron GmbH, 80331 Munich, Germany; 10 Institute of Anatomy, Faculty of Medicine, LMU Munich, 80331 Munich, Germany

**Keywords:** stem cells, adipose tissue derived regenerative cells, ADRCs, point of care treatment, randomized controlled trials, safety, efficacy, stromal vascular fraction, review

## Abstract

It has become practically impossible to survey the literature on cells derived from adipose tissue for regenerative medicine. The aim of this paper is to provide a comprehensive and translational understanding of the potential of UA-ADRCs (uncultured, unmodified, fresh, autologous adipose derived regenerative cells isolated at the point of care) and its application in regenerative medicine. We provide profound basic and clinical evidence demonstrating that tissue regeneration with UA-ADRCs is safe and effective. ADRCs are neither ‘fat stem cells’ nor could they exclusively be isolated from adipose tissue. ADRCs contain the same adult stem cells ubiquitously present in the walls of blood vessels that are able to differentiate into cells of all three germ layers. Of note, the specific isolation procedure used has a significant impact on the number and viability of cells and hence on safety and efficacy of UA-ADRCs. Furthermore, there is no need to specifically isolate and separate stem cells from the initial mixture of progenitor and stem cells found in ADRCs. Most importantly, UA-ADRCs have the physiological capacity to adequately regenerate tissue without need for more than minimally manipulating, stimulating and/or (genetically) reprogramming the cells for a broad range of clinical applications. Tissue regeneration with UA-ADRCs fulfills the criteria of homologous use as defined by the regulatory authorities.

## 1. Introduction: What Are UA-ADRCs and How Are They Used in Regenerative Medicine?

The literature on cells derived from adipose tissue with the aim to apply them in regenerative medicine has become practically impossible to survey, even for experts. A search in PubMed on “adipose derived stem cells” on 22 February 2020 yielded over 10,000 citations, and among them approximately 1000 reviews. Furthermore, among researchers and the public, there still exists some confusion about the terms “pluripotent stem cell”, “multipotent stem cell”, “stromal vascular fraction (SVF)”, “adipose derived regenerative cells (ADRCs)” or “adipose derived stem cells (ASCs)”. For example, a very recent study defined microvascular pericytes as pluripotent adult stem cells with the ability to produce somatic cells typical for the three primitive germ layers (ectoderm, mesoderm and endoderm) [1]. This is in some contrast to, e.g.; the definition of pluripotent stem cells provided by the California Institute for Regenerative Medicine (CIRM) as cells having ‘the potential of taking on many forms in the body, including all of the more than 200 different cell types’ [2]. It is correct that the latter has not been demonstrated for ADRCs and ASCs. On the other hand, the CIRM has defined adult stem cells as cells that ‘are committed to becoming a cell from their tissue of origin, and can’t form other cell types’ [2]. However, we and many others have repeatedly demonstrated that ADRCs and ASCs can indeed form other cell types, because these cells have a three germ layer differentiation capacity [3,4]. These cells are located in the walls of small blood vessels [3]. Since these cells are different from pericytes [3] (as stated in [1]), this adds to the confusion (in a publication considered a milestone in research, these cells were named pericyte/perivascular cells) [5]. 

Many more references regarding characterization of “pluripotent” or “multipotent” stem cells are provided. However, in our opinion it is of equal importance in any study regarding cells in regenerative medicine, to provide a detailed and comprehensive description of the following: (i) the nature of the tissue from which the cells were isolated (this also implies a description of whether the cells are respectively autologous, allogeneic, adult or embryonic or (in experimental studies) xenogeneic), (ii) the isolation procedure itself, including the specific technology that was used, (iii) every process including but not restricted to selecting, cultivating, stimulating, manipulating, (genetically) reprogramming, etc.; to which the cells were exposed during the period between isolation and administration into a patient, (iv) the exact route of administration, including the volume of the administered final cell suspension, (vi) the viability of the cells in a cellular suspension and (vi) every additional therapy that was applied (this comprises any administration of drugs or other biologics such as platelet rich plasma (PRP) before, during or after administration of cells; c.f.; e.g.; [6]). 

In our recent reports about the application of uncultured, autologous, fresh, unmodified, adipose derived regenerative cells (UA-ADRCs) isolated at point of care (i.e.; at the same intervention procedure between harvesting, processing of adipose tissue and injection of cells) with the Transpose RT /Matrase System (InGeneron, Houston, TX, USA), we applied these considerations [7,8,9]. As such, we define treatments with UA-ADRCs as follows: 

Firstly, UA-ADRCs are isolated at the point of care from the patient’s own adipose tissue, usually harvested by a mini-liposuction (in specific cases adipose tissue can also be harvested by surgical extraction). This clearly differentiates UA-ADRCs from cells that are isolated from respectively bone marrow, umbilical cord tissue, umbilical cord blood or from specific organs (such as the isolation of stem cells from tendons, other connective tissue or amniotic or synovial fluid [10]).

Secondly, UA-ADRCs are isolated from adipose tissue such that they are separated from both adipocytes and the connective tissues. In general, one has to differentiate between methods for generating so-called Nanofat (described in the literature as mechanically emulsified fat tissue in a liquid form, ideally devoid of connective tissues but containing cells of the stromal vascular fraction [11]) and methods for isolating only the stromal vascular fraction (i.e.; a cellular extract made from fat that is devoid of both adipocytes and connective tissues [4]). The latter can be achieved with or without the use of enzymes, with a significantly higher cell yield with enzymatic methods (number of nucleated cells per unit weight of adipose tissue or volume of lipoaspirate) than achieved with non-enzymatic methods [4]. Cells that are isolated from adipose tissue in a manner that they are devoid of adipocytes but not of connective tissues (e.g.; [12]) should not be known as stromal vascular fraction and/or ADRCs. 

Thirdly, UA-ADRCs are labelled as *not* or only minimally manipulated if they are *not* cultivated, selected, stimulated, (genetically) or reprogrammed. They can be administered “On Site” into a patient’s damaged tissue in need of regeneration (e.g.; bone defects [7], heart tissue with impaired function as a consequence of previous myocardial infarction [8] or partial tendon ruptures [9], respectively) immediately after isolation of the cells (usually within less than two hours after harvesting of the adipose tissue). Cultivating UA-ADRCs (generating so called ASCs) comes along with possibly culture-related issues affecting their safety as a medicinal product [13]. In addition, culturing and growing cells may reduce their life span by shortening the telomeres following repetitive cell divisions.

Fourthly, we administer UA-ADRCs locally according to the individual patient’s need. In case of bone defects, UA-ADRCs can be administered alone or together with a scaffold [7]. For treating heart failure, we recently published a novel procedure for retrograde administration of UA-ADRCs through the heart’s venous system. Combined with a temporary blockage of the coronary vein at the level of a previous arterial occlusion, this allows application precisely into the cardiac tissue in need of regeneration [8]. In the case of partial tendon ruptures the cells can be directly injected into the damaged site of the tendon [9]. It is obvious that the latter applications require a final cell suspension of small volume, which is achieved with our technology (e.g.; 5 mL of ADRCs in [9]).

Finally, we do not apply any other treatment together with UA-ADRCs, except for adequate rehabilitation (such as optional outpatient rehabilitation with physical therapy modalities in case of tendon regeneration [9]).

In the following text, we present and discuss Eight statements about UA-ADRCs (as defined above) and their application in regenerative medicine, reflecting the current state of knowledge in the literature. They are summarized in Table 1.

## 2. Why and How Regenerative Cells Should Be Isolated from Adipose Tissue Rather Than from Other Tissues, and How These Cells Are Characterized

### 2.1. Statement #1: ADRCs Are Neither ‘Fat Stem Cells’ nor Could They Exclusively be Isolated from Adipose Tissue, as ADRCs Contain the Same Adult Stem Cells That are Ubiquitously Present in the Walls of Small Blood Vessels

One of the greatest misconceptions in stem cell-based regenerative medicine may be the designation of ADRCs as ‘fat stem cells’ and related descriptions in the recent literature (e.g.; ‘adipose-derived stem cells: fatty potentials for therapy’ [14], ‘using fat to fight disease’ [15], ‘adipose tissue stem cells for therapy’ [16] and ‘stem cells derived from fat’ [17]). In fact, these cells are not ‘fat stem cells’ at all. Rather, they are adult stem cells with a three germ layer differentiation capacity located in the walls of small blood vessels (henceforth vascular associated mesenchymal stem cells (MSCs)) (reviewed in [1,4]). Because blood vessels are stimulated to grow, branch and invade developing tissues and organs very early during human embryonic development (starting on approximately day 18 [18]), the presence of vascular associated MSCs in the vascular location results in equal distribution of these cells throughout the body. As a result, vascular associated MSCs in principle can be isolated from small blood vessels in organs other than adipose tissue (shown for heart and skeletal muscle in [3]; strong support for vascular associated MSCs equally distributed in the human body was demonstrated in [5].

The reason why vascular associated MSCs are isolated from adipose tissue is that the latter contains a large amount of small blood vessels and is relatively easy to be harvested in most patients through liposuction. Furthermore, vascular associated MSCs can represent up to 12% of the total population of SVF cells [8], whereas only 0.001%–0.1% of the total population of bone marrow nucleated cells represent MSCs [19,20]. Besides this, harvesting adipose tissue (by liposuction) is typically less invasive than harvesting bone marrow [14,19,21]. Approximately 400,000 elective full liposuction surgeries are performed in the U.S. per year [22], with a serious adverse event rate reported between 0.07% and 0.7% [23,24]. To recover ADRCs, only a mini liposuction of 100 g fat is required. 

Another misconception is the belief that microvascular pericytes represent the vascular associated MSCs in the walls of small blood vessels (e.g.; [1,25,26]). This misconception is based on the fact that expression of the proteoglycan neural/glial antigen 2 (NG2) has long been associated with pericytes [25,26,27]. In the central nervous system (CNS), NG2-positive cells are responsible for the generation of oligodendrocytes [28]. Some authors presented results suggesting that even astrocytes and neurons may be generated from NG2-positive cells, which would make the latter similar to neural stem cells [28]. However, other authors could not reproduce these findings [29]. 

It might be mentioned that different populations of pericytes and pericyte-like progenitor cells are described in the literature. Most probably the best studied pericytes are parts of typical capillary structures, formed by pericytes and endothelial cells [30,31,32]. Another type of pericytes is located at the surface of small blood vessels, partly taking over regulation of vessel diameter and, thus, hemodynamic regulation in the CNS [33]. Pericyte marker expressing cells were described as well in the adventitia of larger vessels [34]. 

**Figure 1:**
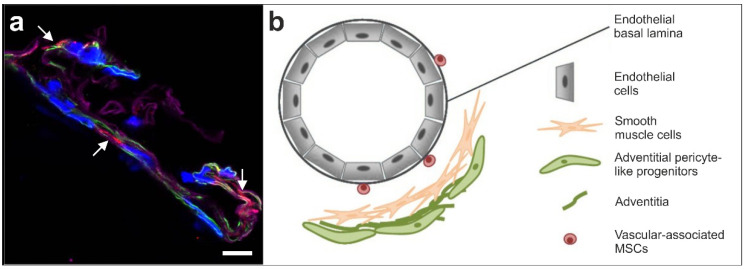
(**a**) Immunofluorescent detection of NG2 (red; arrows), smooth muscle antigen (SMA) (green) and laminin (purple) in the wall of a small arteriole; cell nuclei are in blue (modified from [3]). The scale bar represents 10 µm. (**b**) Schematic illustration of the hypothesized location of vascular associated MSCs (taken from [3] and modified from [35]).

However, all these types of pericytes representing fully matured and differentiated cells are distinct from NG2-positive cells in the wall of a small human arteriole as shown in Figure 1a. This indicates that NG2 or 3G5—considered to be pericyte markers—are expressed by more cells than just the typical pericyte. It is very likely that the early small stem cells share their immunopositivity for NG2 with capillary pericytes [3]. Figure 1b shows the current concept of the localization of vascular associated small early stem cells with a three germ layer differentiation capacity located in the wall of a small vessel.

### 2.2. Statement #2: The Specific Isolation Procedure Used has a Significant Impact on Number and Viability of the Cells Recovered and Hence on Safety and Efficacy of UA-ADRCs

**Figure 2:**
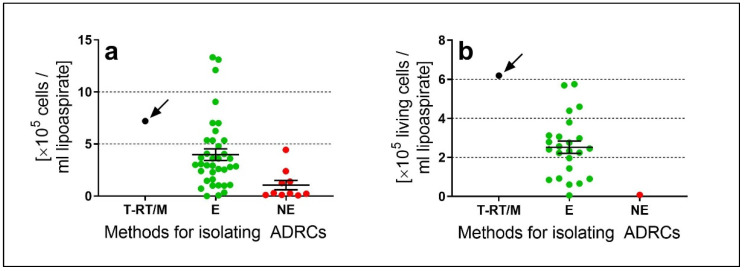
Comparison of cell yield (**a**) and number of living cells per mL lipoaspirate (**b**) of adipose derived regenerative cells (ADRCs) isolated with the Transpose RT/Matrase system (InGeneron) (T-RT/M; black dots) as well as other enzymatic (E; green dots) and non-enzymatic (NE; red dots) methods for isolating ADRCs reported in the literature (modified from [4]). The panels show individual data (dots) as well as mean and standard error of the mean (SEM) in case of the other enzymatic and non-enzymatic methods. One can immediately see that on average enzymatic methods result in a much higher cell yield than non-enzymatic methods. Furthermore, for most of the non-enzymatic methods the number of living cells per mL lipoaspirate could not be calculated because the corresponding relative numbers of living cells were not reported. Of all reported methods the Transpose RT/Matrase system (InGeneron) did not result in the highest cell yield (arrow in a) but in the highest number of living cells per mL lipoaspirate (arrow in b), which appears to be the clinically most relevant parameter (as outlined in the main text).

It is important for physicians and patients to understand that terms such as ‘UA-ADRCs’, ‘fat stem cells’, ‘stromal vascular fraction’, ‘SVF’ etc.; are only generic terms for cell preparations which are isolated from the patient’s own adipose tissue; immediately before transplantation into the target tissue. In fact, an optimal technology for isolating ADRCs should be able to isolate the highest possible number of living ADRCs in the shortest possible time from the smallest possible amount of adipose tissue/lipoaspirate, and should result in the highest possible concentration of cells in the final cell suspension (i.e.; the smallest possible volume of cell suspension) for immediate application to the patient the cells were isolated from. Various enzymatic and non-enzymatic methods were developed for this purpose, some of which are available on the market (reviewed in [4,36,37,38,39]). By using enzymatic methods, the connective tissue and walls of vascular structures of adipose tissue are dissolved by collagenase and the true stem cells can be released by a protease, on average resulting in a cell yield (i.e.; number of isolated ADRCs) significantly higher than when cell are isolated purely mechanically with non-enzymatic methods [4] (Figure 2a). In addition, for the vast majority of non-enzymatic methods described in the literature, no data are provided on the relative number of viable cells in the final cell suspension related to cell death due to the mere mechanical processing of adipose tissue [4]. These considerations are of clinical relevance as (i) the transplantation of an insufficient number of UA-ADRCs might lead to unsatisfactory clinical results. In addition, it does not appear medically justifiable to remove greater amounts of adipose tissue from a patient simply because a non-enzymatic method for isolating ADRCs is used; and (ii) injection of non-viable cells into tissue can lead to inflammatory reactions [40]. 

We recently demonstrated that isolating ADRCs from adipose tissue using the Transpose RT/Matrase System (InGeneron Inc) results in a high cell yield (7.2 × 10^5^ ± 0.90 × 10^5^ ADRCs per mL lipoaspirate in [4]) accompanied by high cell viability (85.9% ± 1.1% in [4]) and, thus an high number of living cells per mL lipoaspirate (6.25 × 10^5^ ± 0.79 × 10^5^ ADRCs per mL lipoaspirate in [4]). To our knowledge the latter is the highest value reported in studies describing methods for isolating ADRCs [4] (Figure 2b). A schematic representation of isolating ADRCs from lipoaspirate with this system is provided in [9].

### 2.3. Statement #3: There is no Need to Further Separate Adipose-Derived Stem Cells (ASCs) from ADRCs if the Latter Were Adequately Isolated from Adipose Tissue

**Figure 3:**
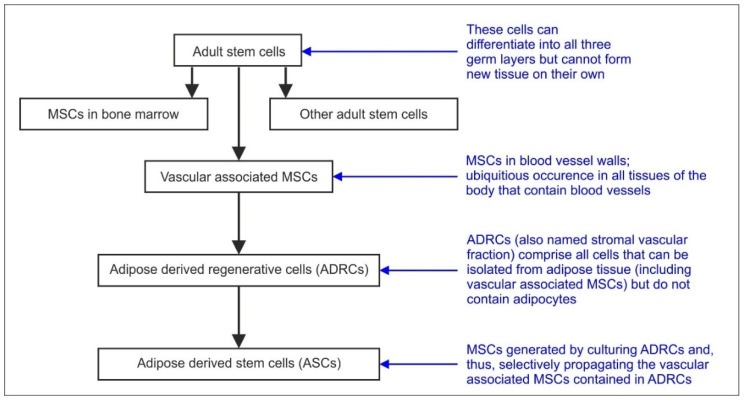
Schematic overview of the relationship between the term adult stem cells, vascular associated MSCs, ADRCs and ASCs (modified from [3]).

Figure 3 provides a schematic overview of the relationship between the terms: adult stem cells, vascular associated MSCs, ADRCs and ASCs. Vascular associated MSCs is a name for a subgroup of adult cells that are contained within ADRCs. ASCs can be obtained by culturing ADRCs and, thus, result from selectively propagating the cells contained in ADRCs. For example, it was shown that culturing ADRCs increased the mean relative number of cells immunopositive for the surface marker (CD29+, a marker in ASCs [39,41]) from 71% at passage 1 to 95% at passage 4, and the mean relative number of CD44+ cells (a marker for fibroblast and progenitor cells [39,41]) from 84% at passage 1 to 98% at passage 4 [42]). However, as both ADRCs and ASCs contain only a relatively small percentage of true stem cells and the other components of the cellular suspension primarily consists of progenitor cells and fibroblasts (characterized by, e.g.; CD 34_+_), the number of true stem cells has been shown to decrease with culturing; as other cells such as fibroblasts grow faster in culture. With culturing in standard culturing media, valuable progenitor cells, such as endothelial progenitor cells (CD34+, CD31+, CD133+), are predominantly lost as well. 

Considering the fact that ASCs also contain cells next to vascular associated MSCs (among them blood-derived cells, endothelial cells and pericytes [8]), one could be inclined to believe that ASCs may be a better choice for tissue regeneration than ADRCs. For the following reasons, however, this might not be the case:

Firstly, cultivating cells inevitably results in exposure of the cells to culture-related mechanic and oxidative factors that have been shown to impact on safety of ASCs as a medicinal product [13]. As a result, ASCs do not meet the criteria of ‘minimally manipulated’ as defined in 21 CFR 1271.10(a) [43] (Title 21 is the portion of the Code of Federal Regulations that governs food and drugs within the U.S. for the FDA [44]) and, thus, may not be regulated solely under Section 361 of the Public Health Service (PHS) Act and 21 CFR Part 1271 [45]. Rather, ASCs may be regulated as a drug, device and/or biologic product under the Federal Food, Drug and Cosmetic (FD&C) Act and/or Section 351 of the PHS Act (and applicable regulations) in the U.S. [45]. The European Medicines Agency considers ASCs as an Advanced Therapy Medicinal Product (ATMP) [46]. 

Secondly, recent in vitro and in vivo studies have indicated non-inferiority or even superiority of UA-ADRCs over ASCs in rescuing heart function after acute myocardial infarction [47] as well as in tendon regeneration [48], bone regeneration [49] or erectile function recovery after cavernous nerve injury [50] (see also [51]). It is currently not clear whether this is due to alterations of the physiological functions of the initial cell suspension when they are (as ASCs) exposed to culture-related factors, or attributable to the mere fact that non cultured ADRCs typically comprise more true stem cells (nuclear Oct4+ cells) than ASCs. This relates to the fact that in cultured ASCs the percentage of true stem cells has been found to be relatively low. Processing of adipose tissue by, i.e.; just using collagenase, results in an initially low content of the very specific stem cells with a three germ layer differentiation capacity in the cellular suspension and, with cell expansion the content of true stem cells is even further reduced. In addition, with every cell division in culturing, the life span and value of the cells decrease further, as in a typical patient requiring stem cell therapy, the individual telomere length of the stem cells is already exhausted and shortened.

### 2.4. Statement #4: The Minimal Definitions of Stem Cells Established by the International Federation for Adipose Therapeutics and Science (IFATS) and the International Society for Cellular Therapy (ISCT) are Somewhat Inadequate and Misleading and Should be Amended

The ISCT published the following minimal criteria for defining multipotent stem cells in 2006: the cells should (i) adhere to plastic, (ii) express the surface markers CD73, CD90 and CD105, and (iii) have the ability to differentiate into chondrocytes, osteoblasts and adipocytes [52]. 

This definition has a number of shortcomings, as the term and function of a true “Stem Cell” with a three germ layer differentiation capacity differs from the term “Progenitor Cell”. Progenitor cells are cells that have already started a differentiation pathway into somatic cells of a certain germ layer, and express the surface markers CD73, CD90 and CD105. But these progenitor cells are not stem cells anymore. Fibroblasts are also adherent to plastic, have the same appearance in cell culture and also express CD73, CD90 and CD105, but are not MSCs as they cannot transdifferentiate into other somatic cells [53]. Of substantial importance however is the fact that the true stem cells with a three germ layer differentiation capacity do not express CD105, CD90 and CD73 [3]. These surface markers are only “later” expressed by progenitor cells. Progenitors are NOT Stem Cells in a correct definition as they are only able to change their differentiation pathway to some degree within their “already started” lineage (such as i.e.; mesoderm), but are not able to differentiate into all three germ layers; as small stem cells with a three germ layer differentiation capacity we find in the blood vessels are able to [3,35]. 

Rather, the expression of cell surface markers is a dynamic process. For example, if MSCs are grown in platelet lysate culture medium or fetal bovine serum, they upregulate different surface markers [3]. Alternatively, they can downregulate surface markers in culture, for example CD34 (a precursor marker) and CD31 (an endothelial precursor marker) expressed previously [3].

Another opinion published by IFATS and ISCT in 2013 on culture-expanded ASCs and SVF stated that the main negative surface markers for these cells are CD45 (<50%) and CD31 (<20%), while the main stable positive surface markers for stromal cells are CD90 (>40)%), CD73, CD44, CD34 (>20%), CD29 and CD13 [54]. In addition, at least 20% of the SVF cells would be CD235a-/ CD45-/ CD34+/ CD31- [54]. 

We recently performed a comprehensive analysis of the relative amounts of ADRCs expressing these surface markers (i.e.; CD90, CD73, CD45, CD44, CD34, CD31, CD29 and CD13) as reported in studies describing enzymatic and non-enzymatic methods for isolating ADRCs [4]. We found that the relative amount of CD34+ ADRCs was determined for only very few methods [42,55,56,57], with substantial variation among these methods (range, 35%–81%). Furthermore, CD34 was determined for most of these methods together with at least one other surface marker, resulting in a large range of published data between 44% (CD34+/CD31-/CD45- cells; [58] and 0.8% (CD34+/CD90-/CD31-/CD45-/CD105-/CD146+ cells; [59]). Similarly, the relative amounts of CD45+ ADRCs varied between 50% [56] and 6% [60] for enzymatic methods, and between 82% [57] and 8% [60] for non-enzymatic methods. Another finding was that the relative amounts of cells positive for CD90, CD73, CD44, CD31, CD29 and CD13 were determined for only a few methods [4]. Most importantly, for none of the published methods the relative amount of CD235a-/ CD45-/ CD34+/CD31- cells was reported, as was proposed by IFATS and ISCT in [54]. Neither was there any correlation between a single surface marker or any combination of surface markers, respectively, on one hand, and the cell yield obtained with a certain method on the other hand [4].

Taken together, the vast majority of reported methods for isolating ADRCs could not be characterized according to the position statements published by IFATS and ISCT [52,54]. Considering the large range of those few data that were reported in this regard, it appears reasonable to hypothesize that determining surface markers of ADRCs is not a suitable method for characterization of stem cells and especially of ADRCs. 

## 3. The Rationale and Advantages of Using Uncultured Autologous Adipose Derived Regenerative Cells (UA-ADRCs) in Regenerative Medicine

### 3.1. Statement #5: Published Peer Reviewed Clinical Research Demonstrates Tissue Regeneration with UA-ADRCs to be Safe and Effective

In a position statement recently published by representatives of the U.S. Food and Drug Administration (FDA) in *The New England Journal of Medicine* [61], safety of stem cell treatment was a primary focus. Marks et al. [61] specifically stated that adverse events are probably more common than is appreciated, because there is no reporting requirement when these therapies are administered outside clinical investigations [61]. In fact, a number of serious adverse events related to stem cell treatments were recently published in *The New England Journal of Medicine* [62,63,64]. These adverse events included the development of a glioproliferative lesion of the spinal cord leading to progressive lower back pain, paraplegia and urinary incontinence after intrathecal infusions of putative mesenchymal, embryonic and fetal neural stem cells for the treatment of residual deficits from an ischemic stroke [62], vision loss after intravitreal injection of autologous ADRCs for the treatment of age-related macular degeneration [63], and lethal human herpesvirus 6–related meningoencephalitis, myocarditis and interstitial nephritis after allogeneic transplantation of stem cells for chronic lymphocytic leukemia [64]. These and other reports about serious adverse events related to stem cell treatments highlight the need to conduct controlled clinical studies in order to determine whether these cellular therapies are safe and effective for their intended uses. Marks et al. [61] concluded that without such studies, one would not be able to ascertain whether the clinical benefits of such therapies outweigh any potential harms. These authors also stated that although autologous stem cells may typically raise fewer safety concerns than allogeneic stem cells, their use may still be associated with adverse events [61] (as demonstrated in [63]).

The outcome of a recent systematic review of reported adverse events in clinical trials on adipose derived cell therapy [65] exemplifies the need to clearly differentiate between the different types of cells derived from adipose tissue, with the aim to apply them in regenerative medicine. The authors of this systematic review identified 70 studies on adipose derived cell therapy involving more than 1400 patients. Twenty out of the 70 studies were used to evaluate thromboembolic safety and mortality, immunological safety and oncological safety. From the nine studies based on which thromboembolic safety and mortality were evaluated, only four were performed with ADRCs, and all of these studies addressed treatment of myocardial infarction (the administration routes were trans-endocardial (two studies), intramyocardial or intracoronary (one study each), respectively). Furthermore, all of the eleven studies based on which immunological safety was evaluated were performed with allogeneic ASCs. In contrast, all the five studies based on which oncological safety was evaluated were performed with ADRCs and did not address musculoskeletal conditions or heart failure. The administration routes in these studies were subcutaneous (two studies), transurethral, periurethral or into the corpus cavernosum of the penis (one study each), respectively. In case of treatments with ADRCs for analyses of thromboembolic safety and mortality as well as oncological safety, no distinction was made between enzymatically and non-enzymatically isolated cells. The authors concluded that adipose-derived cell therapy has so far shown a favorable safety profile, but the safety assessment description has, in general, been of poor quality [65]. Furthermore, they encouraged future studies to maintain a strong focus on the safety profile of cell therapy, so its safeness can be confirmed [65]. The safety of any specific combination of the type of administered cells (enzymatically or non-enzymatically isolated ADRCs, autologous or allogeneic ASCs, etc.), the target tissue and the exact administration route should be evaluated separately. 

For several recent publications reporting therapeutic results of application of fresh, uncultured autologous adipose tissue derived regenerative cells (UA-ADRCs), the safety analysis of [65] is almost irrelevant. These studies included treatment of bone defects [7], of heart failure with retrograde administration of the cells through the heart’s venous system to the area in need of regeneration [8] and of partial tendon ruptures [9]). 

In this regard, we recently performed a prospective randomized, controlled, first-in-human pilot study on safety and efficacy of treating symptomatic, partial-thickness rotator cuff tear (sPTRCT) with UA-ADRCs [9]. Specifically, we treated subjects with symptomatic partial rupture of the supraspinatus tendon with a single injection of UA-ADRCs, and control subjects suffering from the same condition with a single subacromial corticosteroid injection (all patients had not responded to an initial phase of at least six weeks with physical therapy treatments). All injections were made by a qualified physician under ultrasound guidance. Because of its first-in-human character the entire study was carried out according to guidelines set forth by U.S. FDA [45,61]. Over a period of one year after treatment and for all subjects, any illnesses that made it necessary for them to see a physician were documented—completely irrespective of whether the illness that occurred was related to the initial treatment or not (e.g.; the breaking of a tooth in one subject 164 days post treatment). 

This complex procedure prevented reporting of only adverse events that were specifically looked for [65] and resulted, for the first time, in a complete risk profile for the treatment of a musculoskeletal disease with UA-ADRCs [9]. Of note, the risks associated with treatment of sPTRCT with UA-ADRCs were no higher than those with corticosteroid treatment; there were no serious complications related to the treatment. None of the cell treated patients, but one patient treated with corticosteroid injection developed a full rotator cuff tear during the study [9]. This pilot study suggests that the use of UA-ADRCs in subjects with sPTRCT is safe. To verify results of this initial safety pilot study in a larger patient population, a randomized controlled trial on 246 patients suffering from sPTRCT is currently ongoing [66]. 

Furthermore, in a position statement recently published by representatives of U.S. FDA in *The New England Journal of Medicine* [61] it was stated that the literature is replete with instances of therapeutic interventions pursued on the basis of expert opinion and patient acceptance that ultimately proved ineffective or harmful when studied in well-controlled trials comparing them with the standard of care [61]. In this regard another recent systematic review focused on the efficacy of treatments using ADRCs [67]. The authors identified 73 related clinical studies, of which 12 (16.5%) were randomized controlled trials (RCTs) (defined as Evidence Based Medicine (EBM) Level II in [67]), 14 (19.2%) were cohort studies (EBM Level III in [67]) and 47 (64.4%) were case series (EBM Level IV in [67]). Case series and cohort studies are important to determine whether a novel treatment is effective and should be considered for further investigation. However, the only way to reduce certain sources of bias is testing the effectiveness of new treatments in RCTs against no treatment, a conventional treatment or a placebo. We therefore restrict our analysis to the RCTs identified in [67].

Two out of the 12 RCTs listed in [67] might not be considered RCTs in a strict sense. In one of these studies [68] n = 16 subjects with bilateral knee osteoarthritis were treated with cells on one side and with hyaluronic acid (HA) injection on the other side; allocation of either side to cells or HA was performed randomly. In another study, [69] subjects suffering from Achilles tendinopathy were randomly allocated to respectively treatment with cells (n = 21) or treatment with PRP (n = 23). However, for evaluating treatment success using diagnostic ultrasound and magnetic resonance imaging (MRI) all subjects were pooled into one group (n = 44) and no comparisons between the different treatments were performed.

Four other RCTs were excluded from further consideration. In one of them ([70]; focusing on treatment of recalcitrant chronic leg ulcers) centrifuged adipose tissue rather than SVF, ASC or ADRCs was applied. The other three RCTs that were excluded addressed myocardial infarction [71,72,73]. They were excluded because in the APOLLO trial [71] the mean left ventricular ejection fraction (LVEF) was 52% at baseline which is considered an incorrect target population [74]; in the PRECISE trial [72] the LVEF was not investigated with cardiac MRI which is considered the state-of-the-art for accurate, comprehensive and reproducible measurements of cardiac chamber dimensions, volumes, function and infarct size [74]; and the ATHENA trial [73] was initially put on hold because of delivery related cerebrovascular adverse events [7] and afterwards terminated prematurely [73]. 

The remaining six RCTs are summarized in Table 2. In only one of them (addressing Achilles tendinopathy [75]) UA-ADRCs were applied as the sole therapy. The same study was the only one in which a commercially available non-enzymatic) method was used for isolating ADRCs (FastKit; Corios, San Giuliano Milanese, Italy). Only short-term benefits of injecting UA-ADRCs compared to injection of PRP were observed in this study (statistically significantly lower mean VAS pain scores at 15 and 30 days (D15 and D30) post treatment; statistically significantly higher mean VISA-A score on D30 post treatment; and statistically significantly higher mean AOFAS score on D15 post treatment), but no long-term benefits (i.e.; no statistically significantly differences between the groups on D60, D120 and D180 post treatment) [75]. In the other five RCTs listed in Table 2 ADRCs were isolated with experimental, commercially not available (enzymatic) methods, and were not used as the sole therapy. Furthermore, in none of these six RCTs a safety profile comparable to the one established in our recent pilot study on treating sPTRCT with UA-ADRCs [9] was established. 

This overall rather limited evidence base stands in contrast to the high number of enzymatic and non-enzymatic methods for isolating ADRCs that are offered commercially (addressed in detail in Section 2.2). Furthermore, in case of so-called ‘stem cell’ preparations that are recommended, prescribed or delivered in many clinical centers around the world (most probably more than 1000 in the U.S. [81]), these treatments are indeed performed without sufficient data to support their true efficacy [82], supporting the U.S. FDA’s warnings about these stem cell therapies [83]. 

We consider our recent study on safety and efficacy of treating a shoulder tendinopathy with cells ([9] described in detail in Section 3.1) as a first step to provide meaningful data. Patients in the UA-ADRCs cell group showed—both at 24 and 52 weeks post treatment—statistically significantly higher mean ASES total scores (American Shoulder and Elbow Surgeons Standardized Shoulder Assessment Form) than those in the corticosteroid group. The ASES total score takes into account the patient’s pain situation and the functionality of the shoulder [84,85]. A randomized controlled trial on 246 patients suffering from sPTRCT is currently ongoing to verify the results of this pilot study [66].

## 4. How UA-ADRCs Exert Their Functions in Tissue Regeneration

### 4.1. Statement #6: UA-ADRCs Have the Intrinsic Capacity to Adequately Regenerate Tissue without Need for More than Minimal Manipulation, Stimulation and/or (Genetic) Reprogramming of the Cells

In a position statement recently published by representatives of U.S. FDA in The New England Journal of Medicine [61] it was stated that except for a few well-established indications including hematopoietic reconstitution but not including injured knee cartilage and neurologic deficits, there is no scientific evidence supporting claims that stem cells would be intrinsically able to recognize the environment into which they are applied and to determine what kind of tissue must be repaired or replaced [61]. This is in some contrast to profound results from our own published clinical research using UA-ADRCs, as described in the following examples.

**Figure 4:**
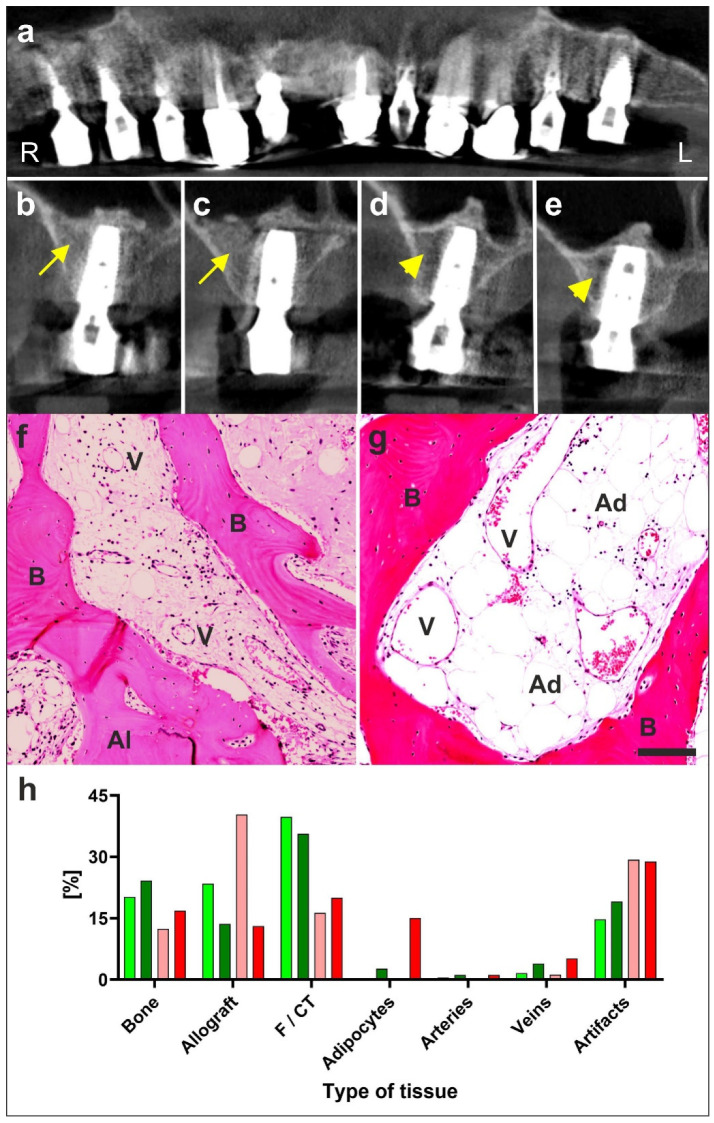
Example of regeneration of bone with UA-ADRCs (modified from [7]). Details are provided in the main text. (**a**–**e**) digital volume tomography radiographs taken 1 year after the placement of implants and 20 months after guided bone regeneration-maxillary sinus augmentation. Panel a provides a panoramic view; Panels (**b**–**e**) show detailed views on selected regions. With the application of unmodified autologous adipose-derived regenerative cells (UA-ADRCs) the bone around the implants in regions B and C appears larger in area and denser (yellow arrows in (b,c)) than without the application of UA-ADRCs in (**d**,**e**) (yellow arrowheads in (**d**,**e**)). (**f**,**g**) Photomicrographs of bone biopsies taken at six weeks (W6) post treatment with application of cells (**f**) or without cells (**g**). (**h**) Results of histomorphometric analysis of bone biopsies taken at W6 (light green and orange bars) and W34 (dark green and red bars) post treatment with application of cells (green bars) or without cells (orange and red bars). Abbreviations: R, right; L, left; B, bone; Al, allograft; V, vein; Ad, adipocyte; F/CT, fibrin and connective tissue. With cells there was considerably more bone and connective tissue formed already at six weeks than was achieved without cells even after six months. The scale bar in g represents 100 µm.

The first example (described in detail in [7]) is a 79-year-old male patient who presented with a partly failing maxillary dentition. This patient was treated with a bilateral external sinus lift procedure and a bilateral lateral alveolar ridge augmentation (henceforth ‘treatment’). On the right side, the treatment was performed with a combination of an osteoinductive scaffold (OIS), fraction 2 of plasma rich in growth factors (PRGF-2) and his own adipose derived regenerative cells (UA-ADRCs, Treatment A). On the left side, the treatment was performed with the same combination of OIS and PRGF-2 but without cells (Treatment B). Then, biopsies were collected at six weeks (W6) and W34 post treatment, and implants were placed at W34. Radiographs that were performed at W32 demonstrated enhanced bone healing with cells (yellow arrows in Figure 4b,c). We did not observe any radiological or histological signs of inflammation. 

Detailed immunohistochemical and histomorphometric analysis of the biopsies demonstrated that Treatment A resulted in faster and better bone regeneration than Treatment B. Specifically, we found even after just 6 weeks significantly more (area/area) newly formed bone, connective tissue and arteries on the right side with cells (Treatment A), than on the left side (Treatment B) after 34 weeks (Figure 4h). Interestingly, a detailed histomorphometric analysis showed a significantly lower relative percentage of adipocytes (2%) with cell treatment than without cells (18%). This is an important finding as it indicates that regenerative cells derived from adipose tissue are primarily not “fat stem cells” only capable of forming adipose tissue, but—as cells with a three germ layer differentiation capacity—capable of adequately forming the needed bone and stroma; while without cells a significantly larger percentage of the newly formed tissue consisted of adipocytes (18%). This might be explained by an initial paucity of locally resident stem cells that were used up in a yearlong inflammatory process of gingivitis, as the typical common basis for tooth loss with age. As the regenerative power of local stem cells is reduced, without new stem cells added, a replacement “Healing” with fat occurs instead.

**Figure 5:**
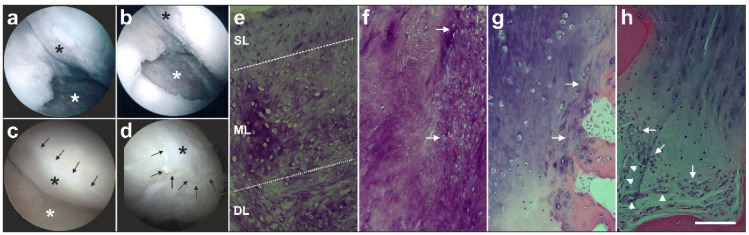
Example of regeneration of knee cartilage with UA-ADRCs (modified from [4]). The letters (**a**–**h**) are explained in detail in the main text above. The scale bar in h represents 100 µm.

The second example (described in detail in [3]) is a male, 51-year-old patient who presented with recurring and increasing pain in both knee joints during walking and other activities. The patient’s history included a tibial chondrocyte transplant that had been performed three years previously. Figure 5a shows an arthroscopic view of third-degree damage to the right tibial plateau where the transplanted chondrocytes had disappeared, and only the artificial matrix with small holes implanted on the tibial plateau was still present (white asterisk in Figure 5a). Furthermore, considerable osteoarthritic damage of the femoral cartilage was observed (black asterisk in Figure 5a). Figure 5b shows the situation after arthroscopic removal of the failed chondrocyte transplant (white asterisk in Figure 5b) as well as ‘mushy’ and damaged cartilage structure on the femoral condyles before it was removed (black asterisk in Figure 5b). Then, the right knee was treated with a single application of UA-ADRCs, whereas the left knee was treated with a standard therapy, i.e.; arthroscopic removal of damaged cartilage and drilling of small holes into the bone. Control arthroscopies were performed one year later. On the right side (treated with UA-ADRCs) complete healing of the tibial defect (white asterisk in Figure 5c) and of the femoral parts (black asterisk in Figure 5c) was observed, with formation of new whitish cartilage that showed a sharp demarcation border to the original, more yellowish cartilage (arrows in Figure 5c). In contrast, a somewhat uneven, overshooting fibroblastic scar formation was found on the left side (treated with a standard therapy) (asterisk in Figure 5d), without the clear and sharp demarcation border to the original cartilage as found with cell treatment (arrows in Figure 5d). This indicated that there was some sort of healing with histologically unorganized fibro-cartilage, but not a regrowth of organized cartilage, as we see it in the right knee after application of UA-ADRCs. Small biopsies that were taken from the regenerated tissue during the follow-up arthroscopies showed the following. After application of UA-ADRCs there was newly formed cartilage with a zonal organization and (like in a textbook of histology) differently shaped chondrocytes in a superficial layer (SL in Figure 5e), middle layer (ML in Figure 5e) and deep layer (DL in Figure 5e). Furthermore, the contact zone between the newly formed cartilage and the bone showed (also like in a textbook of histology) typical chondrocytes with a small nucleus and a hollow space around (arrows in Figure 5f). In contrast, after treatment with a standard therapy there was a more amorphous fibrocartilage with scattered cells (arrows in Figure 5g) but without layered organization, and the contact zone between the newly formed cartilage and bone showed an infiltration of inflammatory cells, fibroblasts (arrows in Figure 5h) and small blood vessels (arrowheads in Figure 5h).

Additional histology results of 14 more patients one year after treatment of knee osteoarthritis 3 and 4 confirm the results of the patient presented above, and are currently prepared for publication.

Accordingly, in both examples, application of UA-ADRCs resulted in better and more adequate tissue regeneration than with standard therapy, as evidenced by immuno-histochemistry and histo-morphometry. Importantly, the beneficial results were achieved in both examples with only minimal manipulation, no stimulation and/or (genetically) reprogramming of the UA-ADRCs prior to transplantation was required. As a result, these examples demonstrate that UA-ADRCs are indeed intrinsically able to sense the environment into which they are introduced and adequately regenerate tissue. Related examples from the fields of wound healing and tendon regeneration can be found in [86,87].

### 4.2. Statement #7: Tissue Regeneration with UA-ADRCs Fulfills the Criteria of Homologous Use

In 21 CFR 1271.3(c) [43], the term homologous use is defined as follows: “repair, reconstruction, replacement, or supplementation of a recipient’s cells or tissues with a human cell, tissue, and cellular and tissue-based product (HCT/P) that performs the same basic function or functions in the recipient as in the donor” [43]. According to FDA’s regulatory considerations for HCT/Ps [45] the replacement of a dysfunctional heart valve is considered homologous use because the donor heart valve performs the same basic function in the recipient as in the donor [45]. In contrast, use of HCT/Ps from adipose tissue for treating musculoskeletal conditions, such as tendon pathologies and arthritis, is primarily not considered homologous use; because promoting the regeneration of tendon or cartilage tissue is not considered a basic function of adipose tissue [45]. 

However, regeneration is not based on adipose tissue as adult tissue, but on the presence of the ubiquitously distributed small universal stem cell in adipose tissue. Based on convincing evidence, regeneration of damaged musculoskeletal tissue is indeed part of the function of specific regenerative stem cells with a three germ layer differentiation capacity contained in UA-ADRCs (and, thus, HCT/Ps from adipose tissue used to treat musculoskeletal conditions can be considered for homologous use). 

Firstly, ADRCs can induce the formation of new blood vessels in adipose tissue [88] as well as in bone [7], ischemic myocardium [8] and other target tissues [89]. Accordingly, application of ADRCs with the aim to induce the formation of new blood vessels fulfills the criterion of the same basic function or functions in the recipient as in the donor and, thus, should be considered homologous use. 

Secondly, it recently has been demonstrated that various pathological conditions result in mobilization of stem cells into the peripheral blood. For example, in the peripheral blood of patients suffering from Crohn’s disease [90] or skin burn injury [91], higher mean numbers of cells expressing markers for endothelial progenitors and stem cells including the very small embryonic-like stem cells (VSELSCs) were found than in the peripheral blood of age-matched controls. Other studies demonstrated mobilization of stem cells into the peripheral blood after acute myocardial infarction in both patients [92] and an animal model [93], and after Achilles tendon transection in an animal model [94]. 

These data raise the question about the origin of these stem cells. In the aforementioned studies on patients suffering from Crohn’s disease or skin burn injury [90,91] VSELSCs were characterized as immunopositive for a number of early true stem cell markers (i.e.; Oct-4+ [95], Nanog+ [96], SSEA-4+ [97] and CXCR4+ [98]) and immunonegative for hematopoietic markers (i.e.; Lin- and CD45- [99]). These VSELSCs also were found in adult bone marrow [100]. Furthermore, Oct-4, Nanog, SSEA-4 and CXCR4 were reported to be also expressed by adipose tissue derived cells [11,100,101,102,103]. Based on our previous research, we therefore consider adipose derived regenerative cells that comprise Oct-4+/Nanog+/SSEA-4+/CXCR4+/CD45- cells to be true stem cells with a three-germ layer differentiation capacity. This is in contrast to the minimal definitions of stromal cells established by IFATS and ISCT ([54]; c.f. Section 2.4.) as their definition does not assume markers such as Oct-4, Nanog, SSEA-4 and CXCR4 to be associated or specific for stem cells.

However, a recent study demonstrated that one day after induction of acute myocardial infarction (AMI) in rats, the number of ASCs was significantly reduced in the stromal vascular fraction compared to healthy control animals, without alterations in the cell surface marker profile and the differentiation capacity of the ASCs [104]. The authors of this study hypothesized that the decreased number of ASCs after AMI could be the result of mobilization of vascular associated MSCs from adipose tissue into the peripheral blood [54,104]. 

Collectively, on the basis of these data one can hypothesize that isolating ADRCs from a patient’s adipose tissue and transplanting these UA-ADRCs into the same patient’s target tissue in need of regeneration (especially when done within the same interventional process) represents augmentation of a physiological process, that also proceeds—but to a lesser extent—on its own. It will be the task of ongoing research to further support these initial findings. In this sense, application of stem cells recovered from adipose tissue for regeneration of musculoskeletal tissue must be considered homologous use, as it is indeed a basic function of a certain component in adipose tissue. 

### 4.3. Statement #8: A Certain Challenge in Research with UA-ADRCs Lays in the Fact that Labeling the Cells Would Render Them Modified, and Unmodified Cells Can Only be Indirectly Identified after Transplantation in a Target Tissue

**Figure 6:**
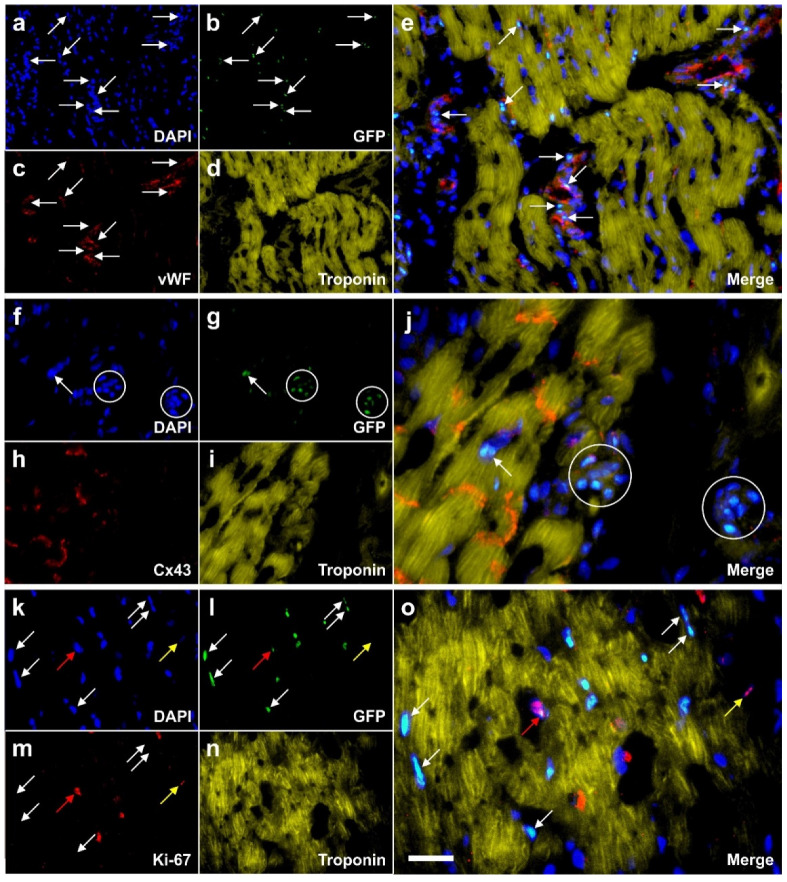
Representative, previously unpublished example how autologous, adipose derived stem cells can stay locally, survive and engraft in the new host tissue into which the cells were applied, differentiate under guidance of the new microenvironment, integrate into the new host tissue and participate in building new vascular structures in the host tissue. The panels show photomicrographs of paraffin-embedded, 5 µm thick tissue sections of a post mortem heart from a pig, taken from the left ventricular border zone of myocardial infarction ten weeks after experimental occlusion of the left anterior descending (LAD) artery for three hours, followed by delivery of eGFP-labeled autologous ASCs into the balloon-blocked LAD vein (matching the initial LAD occlusion site) at four weeks after occlusion of the LAD (experiments are described in detail in [111]). (**a**–**e**) One tissue section was stained with DAPI (blue) (**a**) and processed for immunofluorescent detection of GFP (green) (**b**), von Willebrand factor (vWF) (red) (**c**) and Troponin (yellow) (**d**). The arrows indicate cell nuclei that were immunopositive for GFP and were found in the wall of small vessels (the positions of these cell nuclei are also labeled in the panel representing vWF). (**f**–**j**) Another tissue section was was stained with DAPI (blue) (**f**) and processed for immunofluorescent detection of GFP (green) (**g**), Cx43 (red) (**h**) and Troponin (yellow) (**i**). The circles indicate regions where most of the cell nuclei were immunopositive for GFP, and the arrow a GFP-positive cell nucleus inside (or directly adjacent to) a cardiomyocyte. (k–**o**). A third tissue section was stained with DAPI (blue) (**k**) and processed for immunofluorescent detection of GFP (green) (l), Ki-67 (red) (**m**) and Troponin (yellow) (**n**). The white arrows point to cell nuclei that were immunopositive for GFP but not for Ki-67, the yellow arrows to a cell nucleus that was immunopositive for Ki-67 but not for GFP, and the red arrows to a cell nucleus that was immunopositive for both GFP and Ki-67 (indicating that this cell had re-entered the cell cycle). The scale bar represents 25 µm in the merged panels and 50 µm in the individual panels.

Regarding the potential mechanisms of action of UA-ADRCs in tissue regeneration we should bear in mind that, in contrast to cultured ASCs, unmodified UA-ADRCs in principle cannot be labeled without culturing. Accordingly, without experimental evidence, the determination whether benefits of ASCs also apply to UA-ADRCs must be based on analog conclusions. Specifically, it has been demonstrated that ASCs can stay locally, survive and engraft in the new host tissue into which the cells were applied [105] (a previously unpublished example is shown in Figure 6), differentiate under guidance of the new microenvironment into cells of all three germ layers [4], integrate into and communicate within the new host tissue by forming direct cell-cell contacts [3], exchange genetic and epigenetic information through release of exosomes [3], participate in building new vascular structures in the host tissue [3,7,8] (c.f. Figure 6), positively influence the new host tissue by release of angiogenetic cytokines (among them vascular endothelial growth factor and insulin-like growth factor 1) [106], protect cells at risk in the new host tissue from undergoing apoptosis [106,107,108] and induce immune-modulatory and anti-inflammatory properties [109,110]. The combination of these mechanisms of action render UA-ADRCs a powerful tool in tissue regeneration. 

## 5. Summary

This article demonstrates that well documented basic and clinical research is available to establish a comprehensive understanding for the potential of stem cell therapy with autologous, unmodified UA-ADRCs for widespread application in the practice of medicine. In contrast to multipotent cells that are primarily believed to be able to generate cells of their own germ layer, our stem cells could be defined as “incompletely or partially pluripotent cell that can form cells of all three germ layers but that may not exhibit all the characteristics of completely pluripotent cells” as they have no intrinsic ability and never shown to differentiate into any somatic cells in absence of the right guidance from the microenvironment. This is in striking contrast to embryonic cells, as the adult stem cells strictly depend on signaling from the microenvironment. There has no case of teratoma formation been shown in the literature or clinical studies with adult stem cells.

One important challenge for the near future is to address the substantial discrepancy between the high number of publications on “adipose derived stem cells” in PubMed (>10,000; among them approximately 1000 reviews) on the one hand and the small number of Randomized Controlled Trials (RCTs) published so far. Authorities worldwide including the U.S. FDA and the European EMA will base their judgement about safety and efficacy of tissue regeneration primarily on the results of adequately designed and executed RCTs and/or on convincing clinical follow-up data. As shown here, it is indeed possible to demonstrate safety and efficacy of treatments using UA-ADRCs at a high level of evidence-based medicine. This is of major importance for the benefit of all the patients worldwide in need of effective regenerative therapies. We are consistently asked to ensure that warnings from regulatory authorities about so called “stem cell treatments” based on uncertain variables should one day not be required anymore. 

## Figures and Tables

**Table 1:** Eight statements about uncultured, autologous, fresh, unmodified, adipose derived regenerative cells (UA-ADRCs) and their application in regenerative medicine, reflecting the current state of knowledge in the literature.

Why and How Regenerative Cells Should be Isolated from Adipose Tissue rather than from Other Tissues, and How these Cells are Characterized	
1.	ADRCs are neither ‘fat stem cells’ nor could they exclusively be isolated from adipose tissue, as ADRCs contain the same adult stem cells that are ubiquitously present in the walls of small blood vessels that are capable of differentiating into somatic cells of the three germ layers.
2.	The specific isolation procedure used has a significant impact on the number and viability of the cells and hence on safety and efficacy of UA-ADRCs.
3.	There is no need to further separate adipose-derived stem cells (ASCs) from ADRCs if the latter were adequately isolated from adipose tissue.
4.	The minimal definitions of stem cells established by the International Federation for Adipose Therapeutics and Science (IFATS) and the International Society for Cellular Therapy (ISCT) are somewhat inadequate and misleading and should be amended.
**The Rationale and Advantages of Using UA-ADRCs in Regenerative Medicine**	
5.	Published peer reviewed clinical research demonstrates tissue regeneration with UA-ADRCs to be safe and effective.
**How UA-ADRCs Exert Their Function in Tissue Regeneration**	
6.	UA-ADRCs have the intrinsic capacity to adequately regenerate tissue without need for more than minimal manipulation, stimulation and/or (genetic) reprogramming of the cells.
7.	Tissue regeneration with UA-ADRCs fulfills the criteria of homologous use.
8.	A certain challenge in research with UA-ADRCS lays in the fact that labeling the cells would render them modified, and unmodified cells can only be indirectly identified after transplantation in a target tissue.

**Table 2:** Randomized controlled trials (RCTs) for treating various conditions with UA-ADCRs reported in the literature (c.f. [67]).

Study	Usuelli et al. (2018) [75]	Koh et al. (2014) [76]	Koh et al. (2016) [77]	Han et al. (2010) [78]	Raposio et al. (2016) [79]	Malik et al. (2019) [80]
Indication	Achilles tendinopathy	Knee OA	Knee OA	Skin ulcers	Skin ulcers	Painful amputation stump
N (Tr)	21	26	30	28	16	5
Tr	UA-ADRCs	HTO + UA-ADRCs + PRP	MF + UA-ADRCs	UA-ADRCs + Fibrin	UA-ADRCs + PRP	UA-ADRCs + Fat
I-PoC	Yes	No (1 day before)	No (1 day before)	Yes	Yes	Yes
N (C)	23	26	27	26	24	5
C	PRP	HTO + PRP	MF	Fibrin	PRP	Fat
E/NE	NE	E	E	E	E	E
Follow-up	M6	M24	M26-M30	W8	M18	M6
Tr > C	No	Yes	Yes*	Yes	No**	No

Abbreviations: OA, osteoarthritis; N (Tr), number of subjects in the treatment group; Tr, treatment; HTO, high tibial osteotomy, UA-ADRCs, uncultured, autologous, adipose derived regenerative cells; HTO, open-wedge high tibial osteotomy; PRP, platelet rich plasma; MF, microfractures; I-PoC, isolation of ADRCs at the point of care; N (C), number of subjects in the control group; C, control treatment; E/NE, enzymatic/non-enzymatic isolation of ADRCs; M, month; W, week; Tr > C, outcome after treatment superior to outcome after control treatment. *, only short-term effects (c.f. main text); ** when all subjects were considered that were enrolled in this study.
